# Fine-scale invasion genetics of the quarantine pest, *Anoplophora glabripennis*, reconstructed in single outbreaks

**DOI:** 10.1038/s41598-019-55698-3

**Published:** 2019-12-19

**Authors:** Tetyana Tsykun, Marion Javal, Doris Hölling, Géraldine Roux, Simone Prospero

**Affiliations:** 1 Swiss Federal Research Institute WSL, Zürcherstrasse 111, CH-8903 Birmensdorf, Switzerland; 2 INRA UR633 Zoologie Forestière, CS 40001 Ardon, 45075 Orléans, cedex 2 France; 3 Centre for Invasion Biology, Department of Conservation Ecology & Entomology, Stellenbosch University, Stellenbosch, Republic of South Africa; 4 Université d’Orléans - COST, 45075 Orléans, France

**Keywords:** Invasive species, Population genetics

## Abstract

The xylophagous cerambycid *Anoplophora glabripennis*, the Asian long-horned beetle (ALB), is highly polyphagous and can colonize a wide range of broadleaved host trees causing significant economic damage. For this reason, it is considered a quarantine pest in Europe and North America. Although the global spread of ALB has been depicted recently, no comprehensive studies exist on the genetic pattern of populations’ establishment and dynamics at fine-scale (i.e. within invasive outbreaks), before eradication measures are applied. This information may, however, be particularly important for an efficient management and control of invasive pests. Here, we characterized population genetic diversity and patterns of spread of ALB within and among the four outbreaks detected in Switzerland between 2011 and 2015. For this, we genotyped 223 specimens at 15 nuclear microsatellite loci and conducted specific population-based analyses. Our study shows: (1) At least three independent introductions and a, human-mediated, secondary dispersal event leading to the four outbreaks in the country; (2) An overall low intra-population genetic diversity in the viable and several years active invasive populations; (3) A colonization of single trees by homogeneous ALB genotypes; And (4) an establishment of populations several generations prior to its official discovery.

## Introduction

The intensification of global trade and worldwide mobility has humanitarian and economic benefits, but it also involves potential challenges for native ecosystems due to the introduction of alien species. In recent years, an increasing number of invasive pests and pathogens have been reported in ecosystems worldwide^1–3^. Insights into the species biology and genetic structure of newly established populations, as well as reconstruction of introduction events and invasion routes have the potential to define the factors underlying the invasion success and to outline the pattern of local pests’ outbreaks (e.g. at Janes, *et al*.^4^). This information can be particularly helpful for developing successful management strategies to prevent new invasions and control new outbreaks.

The xylophagous Asian long-horned beetle (ALB; *Coleoptera, Cerambycidae*) *Anoplophora glabripennis* (Motschulsky) is a well-known invasive insect in Europe, in North America, and in artificial plantations in East Asia^5^. ALB can attack a wide range of deciduous trees and survive in cut wood. Trace-back investigations have shown that introductions into Europe and North America occurred via infested wooden packing material from East Asia^6^. In China, the beetle presumably co-evolved in its native range with relict plants like *Eucommia ulmoides*^7^ and, due to reforestation programs in the 1980s, spread as a pest through artificial forests (e.g. plantations, windbreaks) that were frequently established with tree species introduced from Europe (e.g. *Populus*, Smith, *et al*.^8^). In invaded areas, the favourite host trees are species in the genera *Aesculus*, *Acer*, *Betula*, *Fraxinus*, *Platanus*, *Salix*, and *Ulmus*^5^. Female beetles can produce 30–60 (up to 200) eggs annually, which they oviposit in the cambial region of the host trees^9–12^. Although ALB adults, according to laboratory experiments^13,14^, have the potential to fly 14 km, in open environments they fly only 1–3 km during their life span, and when suitable host trees are available most ALB remain in proximity (hundreds of meters) to the tree from which they emerged^6,15^. Larvae feeding on the phloem and xylem tissues, before emerging as young adults from the trees (after 1–2 years), cause the most damage by disrupting the vascular system of the tree^5,16^.

Here, we aimed to investigate the invasion genetics of ALB in the four outbreaks detected in Switzerland between 2011 and 2015 (Table 1). ALB was first found in 2011 on 11 sycamore maples (*Acer pseudoplatanus*) in a private garden in the remote village of Brünisried, Canton Fribourg^9,17^. The source of the infestation could not be determined. During a second survey and sampling in 2013, the presence of exit holes suggested that at least one generation of ALB developed and emerged in Brünisried^17^. In 2012, a new ALB outbreak was found in the industrial area of the city of Winterthur, Canton Zurich^9^. The pest was detected on sycamore maples with new and old exit holes within three infestation spots situated about 250 m apart^9^. In 2014, a large outbreak was detected in Marly, an industrial city situated about 10 km away from the first detected population in Brünisried^18^. In Marly, exit holes were observed on several dozens of trees of *Acer spp*. and *Aesculus hippocastanum*, and ALB was found in all development stages (eggs, larvae, pupae, adults). The infestation area in Marly included two spots (North West and South East), at about 1.5 km distance^17^. The fourth and last Swiss population was detected in Berikon (Canton Aargau) in 2015^19^. A female beetle with fertilized eggs was initially found on a construction site. Later on, a sycamore maple with beetles, egg depositions and exit holes was detected 240 m away from the first finding. Because ALB is an A1 quarantine organism (EPPO 2017), all trees after sampling within outbreaks were eliminated by chipping and/or burning^9^. ALB was also found several times in wooden packaging material at the border of the country and in industrial sites within the country. The relatedness of these beetles to the four outbreaks remained unclear^9^ until the present study.

In this study, we used nuclear microsatellite, i.e. Single Sequence Repeats (SSR), markers to explore the invasion genetics in the four ALB populations described above and in single ALB findings. The SSR markers previously developed specifically for ALB enabled to assess genetic diversity within and among invasive populations and offered a great potential to reconstruct history and the pathways of beetles’ spread using Approximate Bayesian computation analysis^20–22^.

Therefore, with an emphasis on genetic population structure and history of local spread, we addressed the following questions: (1) How many introduction events in Switzerland led to the four outbreaks? (2) What is the genetic relatedness of the beetles in the outbreaks and single findings? (3) How genetically diverse and structured are the invasive populations?

## Results

### COXI and SSR genotyping

Sequencing of the COXI confirmed that all specimens selected for the study belonged to *A. glabripennis*. However, three different COXI haplotypes were observed: specimens from the geographically close outbreaks of Marly and Brünisried showed the COXI haplotype C, whereas specimens from Winterthur and Berikon the haplotype A (Table 2). Single findings were either of COXI haplotype A or B. Of the 223 samples genotyped, 31 showed more than 10% missing data and loci ALB14 and ALB15 were monomorphic. Therefore, we considered 192 multilocus genotypes composed from alleles at 13 SSR loci for the further analysis.

**Table 1:** Summary statistics of the four *Anoplophora glabripennis* outbreaks analysed in the study.

Outbreaks	Year of detection	N. of infested trees	Host species	Development stages	N of specimens
Berikon, Canton Aargau	2015	1	*Acer pseudoplatanus*	imago	7
				pupa	0
				larva	12
				egg	2
Brünisried, Canton Fribourg	2011/2013	11	*Acer pseudoplatanus*, *A. campestre*, *Betula pendula*, *Salix caprea*	imago	0
				pupa	0
				larva	54
				egg	7
Marly, Canton Fribourg	2014	35	*Acer pseudoplatanus, A. campestre, A. negundo, A. platanoides, Betula pendula, Salix caprea, Fraxinus excelsior, Aesculus hippocastanum, Fagus sylvatica, Prunus spp*.	imago	19
				pupa	1
				larva	35
				egg	20
Winterthur, Canton Zurich	2012	55	*Acer pseudoplatanus, A. campestre, Betula pendula, Salix caprea, Populus nigra*	imago	30
				pupa	0
				larva	20
				egg	5

### Population diversity

Locus-specific deviation from HWE and significant correlation between allele frequencies were not consistent among populations (Supplementary Figs. S1 and S2). Allelic diversity per locus within populations was low and in each population, 3–4 loci were monomorphic and the mean number of alleles per locus varied between 1.85 and 2.62 (Table 2). Private alleles were detected in low numbers (up to 4) in all populations and in single findings. All populations except that in Winterthur showed a significant deficit of heterozygosity (H_obs_: 0.19–0.25; H_exp_: 0.26–0.34).

**Table 2:** Quantitative characteristics and summary statistics inferred from 13 nuclear SSR loci of the ALB specimens analysed in this study: specimens, number of individual DNA samples that were analysed; COXI, mitochondrial haplotype inferred from the barcoding region; uMLG, number of unique multilocus SSR genotypes; Na, mean number of alleles per locus (±standard deviation); Pa, number of private alleles; H_exp_, mean expected heterozygosity; H_obs_, mean observed heterozygosity; F_IS_, fixation index (*P valu*e* < 0.05).

Characteristics	Populations				
	Berikon	Brünisried	Marly	Winterthur	Single findings
Specimens	20	53	62	49	7
COXI haplotype	COXI A	COXI C	COXI C	COXI A	COXI A, COXI B
uMLG	20	48	61	45	8
Na	2.08 ± 0.76	2.31 ± 1.18	2.62 ± 1.12	1.85 ± 0.69	—
Pa	1	1	4	0	—
H_exp_	0.33	0.27	0.34	0.26	—
H_obs_	0.22	0.19	0.25	0.25	—
F_IS_	0.32*	0.29*	0.27*	0.01	—

### Population structure

Significant population differentiation (F_ST_ = 0.48–0.59) was observed between all populations pairs, apart from Brünisried and Marly (F_ST_ = 0.07, Table 3).

**Table 3:** Pairwise F_ST_-values between the four ALB populations in Switzerland, inferred from 13 SSR loci.

	Berikon	Brünisried	Marly
Brünisried	0.54*	—	—
Marly	0.48*	0.07	—
Winterthur	0.50*	0.59*	0.52*

*P value < 0.05.

**Figure 1:**
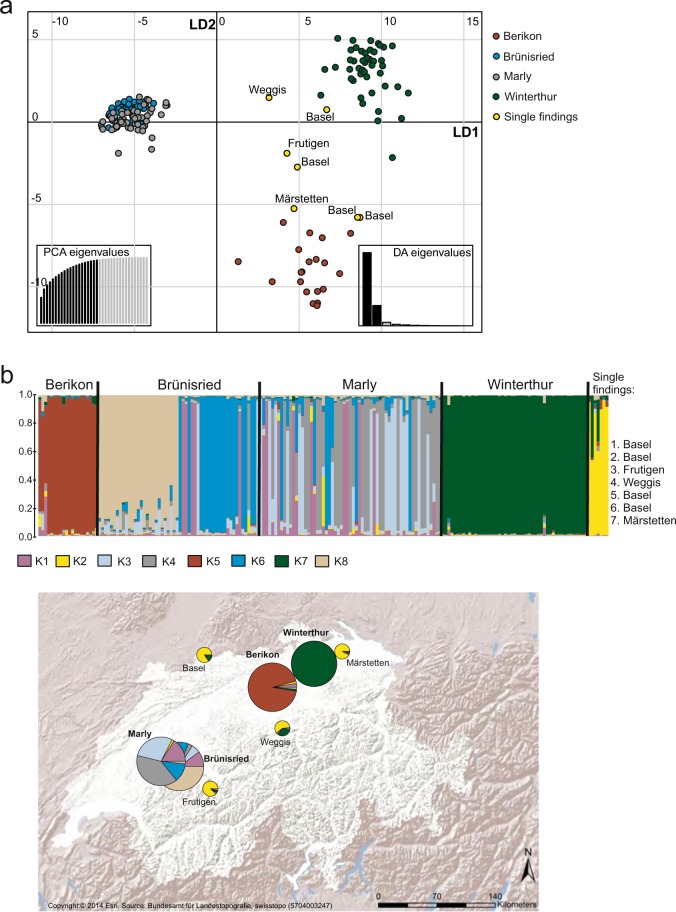
Results of DAPC and Structure analyses based on 192 SSR genotypes from the four ALB populations and individual findings in Switzerland. (**a**) Scatterplot representing the distribution of genotypes (dots) along the first two discriminant axes. Genotypes are colour-coded by population, as indicated on the top of the scatterplot. The PC and discriminant function that were used are represented by dark grey bars in the barplots (bottom left and bottom right insets of the scatterplot, respectively). (**b**) Distribution of ALB and their defined genetic clusters inferred by STRUCTURE analysis. The Structure barplot in the top represents the average estimated probabilities (y-axis) that genotypes belong to specific clusters (K1–K8). Each column represents a different specimen and each colour represents a different cluster as indicated in the bottom from the barplot. The map was generated with ArcGis 10.4.1 using shaded relief background map (Copyright:© 2014 Esri, Source: Federal Office of Topography swisstopo (5704003247)).The pie charts on the map represents estimated probabilities that population (large circle) or single findings (small circle) assigned to specific clusters (K1–K8) as indicated by different colour in the bottom from the barplot.

Overall, multivariate discriminant analysis and Bayesian structure analysis revealed consistent results (Fig. 1a). The DAPC and clustering along the first two discriminant functions showed that ALB specimens from Marly and Brünisried separated clearly from all other specimens along the first discriminant axis LD1 (Fig. 1a). The second axis successfully discriminated Winterthur and Berikon, whereas specimens from Marly and Brünisried overlapped considerably (Fig. 1a). In Berikon and Winterthur, the individual posterior probabilities of assignment to the population of origin were high (>99%) for all samples analysed. On the contrary, in Marly and Brünisried only about 80% of the specimens were clearly assigned to the original population. Based on posterior probabilities, the remaining specimens could be assigned to both populations. In the Structure analysis, considering alteration of assignments in admixed populations (Supplementary Fig. S3) and the second highest difference of the log-likelihood among different K (ΔK peak at K = 8, Supplementary Fig. S3), we assumed eight clusters as reasonable to describe the genetic structure in the four populations (Fig. 1a). The multilocus genotypes from Berikon and Winterthur were clearly assigned to population-specific clusters, suggesting different origin of the two outbreaks and poor genetic diversity of the introduced specimens. By contrast, populations in Marly and Brünisried were characterized by a diverse and admixed genetic structure. ALB genotypes of these two populations were assigned to four common clusters (K1, K3, K4, K6). In addition, about 50% of the genotypes from Brünisried were attributed to a cluster (K8) which was unique for this population. Cluster assignment with both methods showed no clear association of single findings with any of the four Swiss populations.

### Populations and invasion history in marly and brünisried

The population structure analyses described above suggests that populations in Marly and Brünisried were clearly interconnected and substantially differ from the other two Swiss populations (F_ST_ = 0.07, Table 3). Furthermore, the outbreak in Marly incorporate two interconnected populations, that is Marly #1 and Marly #2. Pairwise F_ST_-values showed the largest genetic differentiation between Brünisried and Marly #2 (Table 4). Noteworthy, the F_ST_–value between the spatially close populations Marly #1 and Marly #2 (F_ST_Ma1Ma2_ = 0.12) was 1.5-fold higher than between the geographically more distant (~ 10 km apart) Marly #1 and Brünisried (F_ST_Ma1Br_ = 0.08).

**Table 4:** Pairwise F_ST_–values between the ALB population in Brünisried and the two subpopulations in Marly, inferred from 13 SSR loci.

	Marly #1	Marly #2
Marly #2	0.12*	—
Brünisried	0.08*	0.14*

*P value < 0.05.

**Figure 2:**
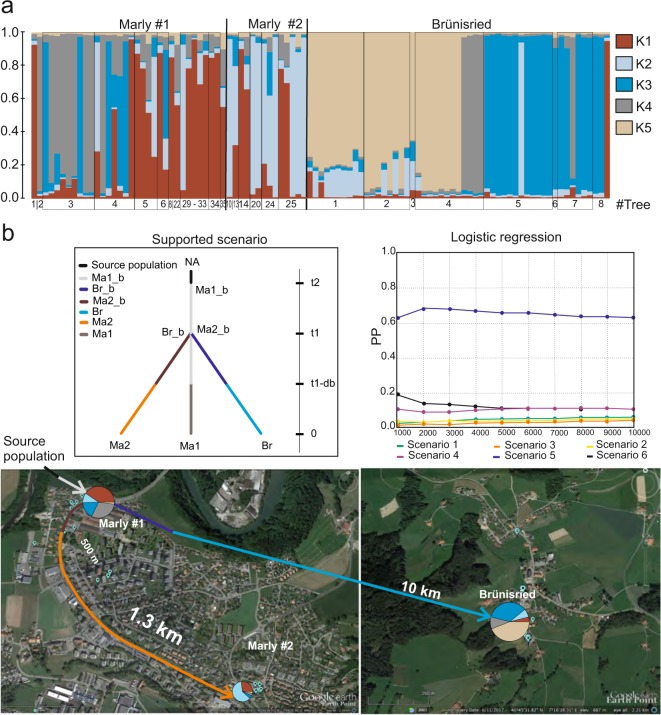
Genetic structure and demographic history of the ALB populations Marly #1, Marly #2 and Brünisried. (**a**) Structure barplot representing the average estimated probabilities (y-axis) that genotypes belong to specific clusters (K1–K5). Each column represents a different specimen and each colour represents a different cluster. The number of trees from which specimens were collected is given below the columns. (**b**) Graphic representation of the ABC analysis results. The diagram on the upper left side shows the demographic events, i.e. change in the effective population size (coloured segment) and corresponding relative time (right vertical scale). Populations of the Marly #1 area are designated as Ma1_b and Ma1, of the Marly #2 area as Ma2_b and Ma2, and of the Brünisried area as Br_b and Br. The graphic on the upper right side indicates the relative posterior probabilities of competing scenarios. The two scheme on satellite images at the bottom of the figure illustrate the spatial spread of ALB according to the supported scenario. The satellite images with location marks were generated using online web pages www.earthpoint.us and Google Earth V 7.1.5.1557, images sources: Google, Image Landsat/Copernicus; the first image from the left: 9/28/2018, Marly, Switzerland, 46°46′54.93″ N 7°09′20.96″ E, elev 651 m, Eye alt 2.16 km; the second image: 6/11/2017, Brünisried, Switzerland, 46°45′31.82″ N 7°16′38.31″ E, elev 887 m, Eye alt 2.21 km.

Following the same approach as described above in the Structure analysis, we inferred K = 5 as the number of genetic clusters that best explain the population in Marly #1, Marly #2 and Brünisried (Fig. 2a, Supplementary Fig. S4). In Brünisried most genotypes were assigned to a single cluster, whereas in both populations in Marly admixed genotypes were frequent. In all populations considered, ALB genotypes originating from a single tree were mostly attributed to a single cluster. In Brünisried, about 50% of the genotypes (trees 1 to 4 in Fig. 2a) were assigned to the K5 cluster (sand colour, Fig. 2a), which is unique to this population, while the rest were assigned to the clusters K4 (grey), K3 (blue) and K1 (red), which were also common in Marly #1.

Considering the F_ST_-values between population pairs (F_ST_Ma1Br_ < F_ST_Ma1Ma2_ < F_ST_Ma2Br_) and the Structure results, six different scenarios of the demographic history were tested for the ABC analysis (Supplementary Fig. S5). The highest posterior probabilities (Table 5) with non-overlapping 95% Cis, inferred from 500 and 1% of simulated data closest to the observed using a direct approach and a linear discriminant transformation of the summary statistic values accordingly, were computed for the fifth scenario (see other details in Supplementary). The second ABC step supported the same scenario (Table 5, Fig. 2b, parameters’ estimations see in Supplementary Table S2). This specific scenario assumes that an initial ALB population of a small effective size arrived to Marly #1 about 8–10 generations ago from an unknown source population (NA). From the establishing population Ma1_b with an estimated effective population size of 36 individuals (here and after mode values were considered, Supplementary Table S2), some beetles reached Marly #2 and Brünisried at approximately the same time (presumably 7–8 generations ago) and founded two new populations of limited effective sizes (estimated 13 specimens in Ma2_b and 31 specimens in Br_b, Supplementary Table S2). Thereafter, populations developed to the sampled populations in Marly #2 and Brünisried (Ma2 and Br in Fig. 2b). At the same time, presumably over 6–7 generations, the initial population (Ma1_b) in Marly #1 independently developed to the sampled population Ma1 (Fig. 2b).

**Table 5:** Mean posterior probabilities of the competing models for ABC analysis.

Post. prob. 0.95 CI	SC1	SC2	SC3	SC4	SC5	SC6
**First step**						
Direct^a^	0.16 ± 0.01	0.14 ± 0.01	0.14 ± 0.01	0.16 ± 0.01	0.27 ± 0.03	0.13 ± 0.01
LDA^b^	0.05 ± 0.01	0.04 ± 0.01	0.03 ± 0.01	0.11 ± 0.01	0.65 ± 0.02	0.12 ± 0.03
**Second step**						
Direct^1^	0.61 ± 0.01	0.39 ± 0.01	—	—	—	—
LDA^2^	0.71 ± 0.04	0.29 ± 0.04	—	—	—	—

^a^Posterior probabilities inferred directly from summary statistics of the 500 closest to observed simulated data sets

^b^Posterior probabilities inferred from linear discriminants of the summary statistics of the from 1% of 10^6^ simulated datasets.

## Discussion

### Invasion genetics and history of ALB outbreaks

To our knowledge, our study is the first to focus on the genetic structure of ALB at a very fine scale. The ALB populations considered in this study are characterized by an overall low genetic diversity, similar to that reported with the same microsatellite markers for other invasive populations, e.g. in North America and Europe^20,22,23^.

The high differentiation and contrasting patterns of genetic population structure strongly support three independent introduction events lead to the four Swiss populations. Specifically, the two populations in Berikon and Winterthur, were assigned to two distinct clusters, both having no trace in others population in Switzerland. The remaining two populations, i.e. Marly and Brünisried, are substantially different from the others, but share a common genetic pool and computed clusters, suggesting an origin from the same source population. Noteworthy, none of the single findings could be genetically bound to one of the studied populations. This result suggests that single findings most likely originated from independent introductions. In this case, the introduced beetles, for one reason or another (e.g. rapid detection, presence of only males), failed to establish new populations.

The strong population differentiation coupled with deficit of heterozygotes suggests no gene flow between the geographically distant populations, which is expected considering the limited natural dispersal of ALB. As already observed in open environment, when suitable host trees are available most ALBs remain in proximity to the tree from which they emerged^6,15^. Results of our genetic population structure analysis confirm the field observations. In all populations, only one or maximum two genetic clusters were found on a single tree. Remarkably, riparian broadleaf stands that are potentially attractive for the polyphagous ALB exist in a proximity of 140 m to the first infested area (Marly #1). However, the second population (Marly #2) was established at a distance of about 1.3 km from the first, along a main road, which could be explained by local human-mediated transportation (e.g. hitch-hiking).

### Invasion history in Marly and Brünisried

Reconstruction of the invasion history of the interconnected populations of Marly and Brünisried with Approximate Bayesian Computation (ABC) suggests that initially up to two hundred ALB individuals arrived in the industrial area of Marly (Marly #1, Fig. 2 and Ma1_b estimated parameters in Supplementary Table S2), perhaps with several introduction events. Subsequently, beetles might have been transferred to the second infestation area in Marly and to Brünisried. Trace back investigations revealed that firewood originating from the infestation in Marly was transported by a citizen to Brünisried and most likely acted as vector for ALB. Indirect field evidence (e.g. presence of old exit holes) confirms that the population in Marly was established at least four generations before its detection^17^, that would support the oldest origin of the population in Marly. The genotypes with admixed assignments indicate that beetles had probably several generations for breeding prior to sampling. The multiple genetic clusters in the oldest population may be also the result of introductions from a diverse population, presumably in South Korea as suggested in the study of Javal, *et al*.^22^. The presence in the stepwise population of Brünisried of a cluster that was not detected in the initial population of Marly, might be explained either by genetic drift that had occurred, or by the introduction of specimens from another, unidentified population. However, considering that i) Brünisried is a relatively small and remote village and ii) there is no evidence of such an unknown population in the surrounding region, we think that the last explanation is unlikely. As expected, the founder population (Marly #1) was most likely of limited effective size, owing to a transitory bottleneck. According to the supported ABC scenario and the estimated parameters, this first population experienced a lag invasion phase, i.e. a period of low growth and reduced effective size^24^. Intriguingly, this period was probably twice as long in the initial population as in the step-wise populations. It could be that during this period natural selection acted in favour of the phenotypes which performed best in the new environment. Thus, the beetles that established subsequent populations were already better adapted than those that initially arrived. Despite a possible uncertainty in the specific quantitative results of our ABC analysis, it shows that all three populations with common origin increased their effective sizes in about the same time period and, given also to the high fecundity of female beetle (up to 200 eggs annually^15^), had the potential to restore populations of up to a few thousands specimens. Although, field observations during sampling suggest that the populations in Marly and Bruenisried were less abundant in reality.

## Conclusions

Thanks to continuous monitoring of ALB in the field and sampling during eradication motions, we had the opportunity to analyse a large number of ALB specimens from the four outbreaks observed in Switzerland, and provide new detailed insights into the invasion genetics of this pest at a population level. First, we showed that at least three independent introduction events lead to the four Swiss outbreaks. In addition, eight unrelated single findings suggest additional independent introductions which did not lead to the establishment of populations. This highlights the importance of regular inspections for presence of ALB at potential entry points in wooden material. Second, we confirmed that in the invasive populations, ALB naturally disperses and breeds mainly in a single tree or in trees in close proximity (e.g. Marly and Brünisried). ALB genotypes originating from a single tree were mostly genetically homogeneous and belonged to the same genetic cluster. However, our analyses also revealed an efficient secondary dispersal of ALB within Switzerland, most likely by human activity. Consequently, when a new outbreak of an invasive pest is detected, intensive monitoring should also target trees along the main transportation and communication routes (e.g. roads, railways) near the outbreak. Remarkably, the largest Swiss population (Winterthur) was genetically homogeneous but have been successfully active by several years before eradication. It seems that low genetic diversity does not compromise the ability of ALB to become established outside its native range.

## Methods

### Samples of ALB

A total of 223 ALB specimens were sampled at different development stages and analysed in this study (Table 1). Most of them were collected at the four outbreak areas during the implementation of eradication measures. In addition, four single specimens originated from wooden packaging material from China found in ports around Basel. Two specimens were found at construction sites in Märstetten and Frutigen. One adult male beetle was caught in a car near the village of Weggis (Canton Lucerne, Switzerland). As no infested trees or wooden packing material were detected in the surrounding area, the origin of this beetle remains unclear^9^.

### DNA extraction and species identification

Until DNA extraction, the ALB specimens were preserved in absolute ethanol at −20 °C. DNA extraction was performed using the DNeasy Blood & Tissue Kit (Qiagen, Hombrechtikon, Switzerland), following the manufacturer’s protocol. DNA was extracted from one leg (adults), the head (pupae, larvae) or entire eggs. The barcoding region of the mitochondrial gene cytochrome oxidase I (COXI) was amplified using the standard primer pair HCO2198 and LCO1490^25^. PCR products were purified with Illustra™ ExoProStar™ mix (Sigma Aldrich, St. Louis, MO, USA), following the manufacturer’s instructions. Sequencing reactions were performed at both strands separately, using the Big Dye Terminator sequencing kit (v 3.0, Applied Biosystems, Foster City, CA, USA), on an ABI 3130 Genetic Analyzer at Swiss Federal Research Institute WSL (Birmensdorf, Switzerland). The obtained sequences were edited manually using GeneStudio (TM) Professional Edition 2.1.2.3 and analysed with CLC Main Workbench 7. The sequences were then BLAST searched against the non-redundant nucleotide collections of the NCBI database (https://www.ncbi.nlm.nih.gov/). A COXI haplotype was assigned when match similarity of the BLAST search was greater than 98%.

### Genotyping

Samples were genotyped at 15 microsatellite loci developed by Carter, *et al*.^26^. Amplifications were multiplexed in six reactions as follows: (1) ALB38/ALB77, (2) ALB10/ALB53/ALB59, (3) ALB9/ALB14, (4) ALB15/ALB35/ALB44, (5) ALB40/ALB24, and (6) ALB43/ALB19/ALB30). Multiplexed PCR reactions were performed using the Multiplex PCR Kit (Qiagen), as described by Javal, *et al*.^22^. All PCR amplifications were performed on a Veriti 96-Well Fast Thermal Cycler (Applied Biosystems), and PCR products were subsequently denatured by formamide before fragment sizing on ABI PRISM 3500 (Life Technologies, CA, USA). Allele sizes were scored with the GeneMapper v5 software (Applied Biosystems). Ambiguous loci and genotypes that could not be reliably scored were re-amplified and re-analysed.

### Data analysis

For population genetics analyses in the four outbreaks in Switzerland (Berikon, Brünisried, Marly, Winterthur), each outbreak was considered as a population. In each ALB population, the number of alleles per SSR locus, the observed and expected heterozygosity^27^, the number of private alleles, the evenness of multilocus genotypes^28^, fixation indices, and the deviation from Hardy-Weinberg equilibrium^29^ were estimated using Arlequin 3.5.2.1^30^ and the R-package *poppr* v 2.3.0^31^. Pair-wise linkage disequilibrium (LD) between loci was tested with the log-likelihood ratio using a Markov chain algorithm (default parameters), as implemented in the web version of Genepop 4.2^32^. The statistical significance of LD was inferred using 1000 permutations and a sequential Bonferroni correction with α = 0.05. Genetic differentiation among populations was assessed by calculating pairwise *F*_ST_-values^29^ and corresponding *P* values (α = 0.05) with Arlequin 3.5.2.1.

Genetic relatedness of the ALB specimens among populations as well as single findings was studied with a multivariate clustering method, i.e. discriminant analysis of principle components (DAPC), implemented in the R-package *adegenet*^33^. First, multilocus genetic data were transformed into principal components (PCs) and the optimal number of PCs was determined with cross-validation^33^. Thereafter, ALB groups were predefined according to the origin of the specimens (i.e., the four outbreaks and the single findings). Samples were then plotted along the first two discriminant functions of the analysed PCs.

The population genetic structure of the four outbreaks was analysed with the Bayesian model-based cluster analysis, as implemented in STRUCTURE v 2.3.4. ALB specimens were probabilistically assigned to genetic clusters using allele frequencies at each locus. No prior geographic information was used (LOCPRIOR = 0 option), and the admixture ancestral model with correlated allele frequencies was applied. Analyses were run with 200,000 burn-in iterations, followed by the same number of iterations for Markov chain Monte Carlo (MCMC) in ten independent runs for each number of clusters (K) from 1 to 20. The most likely K was determined, as suggested in Janes, *et al*.^34^, by (1) considering the maximal mean and small standard deviation of the posterior probability of K among runs^35^, (2) applying ΔK methods^36^, using Structure Harvester^37^, and (3) analysing the alterations of individual assignment probabilities with increasing K (i.e. whether additional clusters were represented with a high probability by at least one specimen or whether probabilities rather were portioned among several individuals). Average assignment probabilities of specimens to the genetic clusters were computed with Clumpp 1.1.2^38^ using the greedy algorithm for K ≥ 10 and visualized using Distruct 1.1^39^ and R graphic functions. Spatial distribution of the defined genetic clusters and their probabilistical assignments in populations were mapped with ArcGis 10.4.1.

The demographic history of the ALB spread in the two related outbreaks of Marly and Brünisried (see Results) was investigated using a coalescent approximate Bayesian computation approach implemented in DIYABC v.2.1.0^40^. For this analysis, the two infestation areas (North West and South East spots) in Marly were considered as different populations, that is Marly #1 (34 individuals) and Marly #2 (14 individuals), geographic coordinates for the rest of 14 individuals in Marly were not recorded, thus they were excluded from the analysis. The demographic scenario that best explained the observed genetic diversity in outbreaks was inferred from two analysis steps (for details see Supplementary). First, the most likely sequences of demographic events and defined topology of ALB populations’ spread were tested. In this step, we used a broad frame of prior population parameters to compute simulated data-sets. Then, considering information from the field observations we restricted the first event of the ALB arriving to 10 generations ago and defined the time of each demographic event in a likelihood scenario. In both steps, the ABC analysis was conducted following Cornuet, *et al*.^41^ and included the following steps: (1) assume realistic competing scenarios considering structure, F_ST_ ratio between sampled populations, and field observations; (2) simulate 1 × 10^6^ pseudo-observed datasets (PODs) for each scenario and compute correspondent summary statistics; (3) evaluate posterior probabilities of each scenario on 1% PODs with the closest summary statistic to the observed dataset and identify the best scenario in 95% confidence interval; (4) assess the confidence level of the chosen scenario as the proportion of times that this scenario was falsely rejected (type-I error) or accepted (type-II error); (5) evaluate the goodness-of-fit of the selected scenario to the data.

### Ethical approval

The article does not contain any studies with human participants or vertebrate animals.

## Supplementary information

Supplementary information

## Data Availability

Genotype data submitted to Dryad 10.5061/dryad.dfn2z34wc.

## References

[1] Fisher MC (2012). Emerging fungal threats to animal, plant and ecosystem health. Nature.

[2] Liebhold AM, Brockerhoff EG, Nuñez MA (2017). Biological invasions in forest ecosystems: a global problem requiring international and multidisciplinary integration. Biological Invasions.

[3] Lowe, S., Browne, M., Boudjelas, S. & De Poorter, M. *100 of the world’s worst invasive alien species: a selection from the global invasive species database*. Vol. 12 (Invasive Species Specialist Group Auckland, 2000).

[4] Janes JK (2015). Polygamy and an absence of fine-scale structure in Dendroctonus ponderosae (Hopk.) (Coleoptera: Curcilionidae) confirmed using molecular markers. Heredity.

[5] Haack RA, Hérard F, Sun J, Turgeon JJ (2010). Managing invasive populations of Asian longhorned beetle and citrus longhorned beetle: a worldwide perspective. Annual review of entomology.

[6] Hu J, Angeli S, Schuetz S, Luo Y, Hajek AE (2009). Ecology and management of exotic and endemic Asian longhorned beetle Anoplophora glabripennis. Agricultural and Forest Entomology.

[7] Pan L, Wang R, Zhang Y-R, Feng Y-Q, Luo Y-Q (2015). Antifeedant activity of gutta-percha against larvae of the Hyphantria cunea and Anoplophora glabripennis. Journal of Plant Interactions.

[8] Smith MT, Turgeon JJ, De Groot P, Gasman B (2009). Asian Longhorned Beetle Anoplophora glabripennis (Motschulsky): Lessons Learned and Opportunites to Improve the Process of Eradication and Management. American Entomologist.

[9] Forster B, Wermelinger B (2012). First records and reproductions of the Asian longhorned beetle Anoplophora glabripennis (Motschulsky) (Coleoptera, Cerambycidae) in Switzerland. Mitteilungen der Schweizerischen Entomologischen Gesellschaft = Bulletin de la Société Entomologique Suisse = Journal of the Swiss Entomological Society.

[10] Meng PS, Hoover K, Keena MA (2015). Asian Longhorned Beetle (Coleoptera: Cerambycidae), an Introduced Pest of Maple and Other Hardwood Trees in North America and Europe. Journal of Integrated Pest Management.

[11] Keena MA (2002). Anoplophora glabripennis (Coleoptera: Cerambycidae) Fecundity and Longevity Under Laboratory Conditions: Comparison of Populations from New York and Illinois on Acer saccharum. Environmental Entomology.

[12] Keena MA (2006). Effects of Temperature on Anoplophora glabripennis (Coleoptera: Cerambycidae) Adult Survival, Reproduction, and Egg Hatch. Environmental Entomology.

[13] Lopez VM, Hoddle MS, Francese JA, Lance DR, Ray AM (2017). Assessing Flight Potential of the Invasive Asian Longhorned Beetle (Coleoptera: Cerambycidae) With Computerized Flight Mills. Journal of Economic Entomology.

[14] Javal M, Roux G, Roques A, Sauvard D (2018). Asian Long‐horned Beetle dispersal potential estimated in computer‐linked flight mills. Journal of Applied Entomology.

[15] Smith MT, Bancroft J, Li G, Gao R, Teale S (2001). Dispersal of Anoplophora glabripennis (Cerambycidae). Environmental Entomology.

[16] Javal Marion, Roques Alain, Haran Julien, Hérard Franck, Keena Melody, Roux Géraldine (2017). Complex invasion history of the Asian long-horned beetle: fifteen years after first detection in Europe. Journal of Pest Science.

[17] Fragnière Y, Forster B, Hölling D, Wermelinger B, Bacher S (2018). A local risk map using field observations of the Asian longhorned beetle to optimize monitoring activities. Journal of Applied Entomology.

[18] Meier F (2015). Forstschutz-Überblick 2014. WSL Berichte, Birmensdorf, Eidgenössische Forschungsanstalt für Wald, Schnee und Landschaft.

[19] Meier F (2016). Waldschutz-Überblick 2015. WSL Berichte, Birmensdorf, Eidgenössische Forschungsanstalt für Wald, Schnee und Landschaft.

[20] Carter ME, Smith MT, Harrison RG (2009). Patterns of Genetic Variation among Populations of the Asian Longhorned Beetle (Coleoptera: Cerambycidae) in China and Korea. Annals of the Entomological Society of America.

[21] Carter M, Smith M, Harrison R (2010). Genetic analyses of the Asian longhorned beetle (Coleoptera, Cerambycidae, Anoplophora glabripennis), in North America, Europe and Asia. Biological Invasions.

[22] Javal Marion, Lombaert Eric, Tsykun Tetyana, Courtin Claudine, Kerdelhué Carole, Prospero Simone, Roques Alain, Roux Géraldine (2019). Deciphering the worldwide invasion of the Asian long‐horned beetle: A recurrent invasion process from the native area together with a bridgehead effect. Molecular Ecology.

[23] Carter ME, Smith MT, Turgeon JJ, Harrison RG (2009). Analysis of genetic diversity in an invasive population of Asian long-horned beetles in Ontario, Canada. The Canadian Entomologist.

[24] Whitney KD, Gabler CA (2008). Rapid evolution in introduced species, ‘invasive traits’ and recipient communities: challenges for predicting invasive potential. Diversity and Distributions.

[25] Folmer O, Black M, Hoeh W, Lutz W, Vrijenhoek R (1994). DNA primers for amplification of mitochondrial cytochrome c oxidase subunit I from diverse metazoan invertebrates. Molecular marine biology and biotechnology.

[26] Carter M, Casa AM, Zeid M, Mitchell SE, Kresovich S (2009). Isolation and characterization of microsatellite loci for the Asian longhorned beetle, Anoplophora glabripennis. Mol. Ecol. Resour..

[27] Nei M (1978). Estimation of average heterozygosity and genetic distance from a small number of individuals. Genetics.

[28] Grünwald NJ, Goodwin SB, Milgroom MG, Fry WE (2003). Analysis of genotypic diversity data for populations of microorganisms. Phytopathology.

[29] Weir B. S., Cockerham C. Clark (1984). Estimating F-Statistics for the Analysis of Population Structure. Evolution.

[30] Excoffier L, Hofer T, Foll M (2009). Detecting loci under selection in a hierarchically structured population. Heredity.

[31] Kamvar ZN, Tabima JF, Grunwald NJ (2014). Poppr: an R package for genetic analysis of populations with clonal, partially clonal, and/or sexual reproduction. PeerJ.

[32] Rousset F (2008). genepop’007: a complete re-implementation of the genepop software for Windows and Linux. Mol. Ecol. Resour..

[33] Jombart T, Devillard S, Balloux F (2010). Discriminant analysis of principal components: a new method for the analysis of genetically structured populations. BMC Genet.

[34] Janes JK (2017). The K = 2 conundrum. Mol Ecol.

[35] Pritchard JK, Stephens M, Donnelly P (2000). Inference of population structure using multilocus genotype data. Genetics.

[36] Evanno G, Regnaut S, Goudet J (2005). Detecting the number of clusters of individuals using the software STRUCTURE: a simulation study. Molecular ecology.

[37] Earl DA, vonHoldt BM (2011). Structure Harvester: a website and program for visualizing Structure output and implementing the Evanno method. Conservation Genetics Resources.

[38] Jakobsson M, Rosenberg NA (2007). CLUMPP: a cluster matching and permutation program for dealing with label switching and multimodality in analysis of population structure. Bioinformatics.

[39] Rosenberg N (2004). A. distruct: a program for the graphical display of population structure. Molecular Ecology Notes.

[40] Cornuet J-M (2014). DIYABC v2.0: a software to make approximate Bayesian computation inferences about population history using single nucleotide polymorphism, DNA sequence and microsatellite data. Bioinformatics.

[41] Cornuet J-M, Ravigné V, Estoup A (2010). Inference on population history and model checking using DNA sequence and microsatellite data with the software DIYABC (v1.0). BMC Bioinformatics.

