# Supplementary material for: Fine-scale invasion genetics of the quarantine pest, Anoplophora glabripennis, reconstructed in single outbreaks
Source: Sci Rep. 2019 Dec 19;9:19436. doi: 10.1038/s41598-019-55698-3 (PMC6923442; doi:10.1038/s41598-019-55698-3)
Supplement: Supplementary file 1 — Supplementary information [file 41598_2019_55698_MOESM1_ESM.pdf]

Fine-scale invasion genetics of the quarantine pest, *Anoplophora glabripennis*, reconstructed in single outbreaks

Tetyana Tsykun[1,*], Marion Javal[2,3], Doris Hölling[1], Géraldine Roux[2,4], Simone Prospero[1]

[1] Swiss Federal Research Institute WSL, Zürcherstrasse 111, CH-8903 Birmensdorf, Switzerland;
[2] INRA UR633 Zoologie Forestière, CS 40001 Ardon, 45075 Orléans cedex 2, France;
[3] Centre for Invasion Biology, Department of Conservation Ecology & Entomology, Stellenbosch University, South Africa;
[4] Université d'Orléans - COST, 45075 Orléans, France.

*Correspondent author: Dr. Tetyana Tsykun,
Swiss Federal Research Institute WSL,
Zürcherstrasse 111, CH-8903 Birmensdorf,
Switzerland,
e-mail: tetyana.tsykun@gmail.com

Supplementary information

Figure S1. Deviation from Hardy-Weinberg equilibrium in 13 SSR loci. Red color indicates *P* values < Bonferroni corrected threshold with α=0.05.

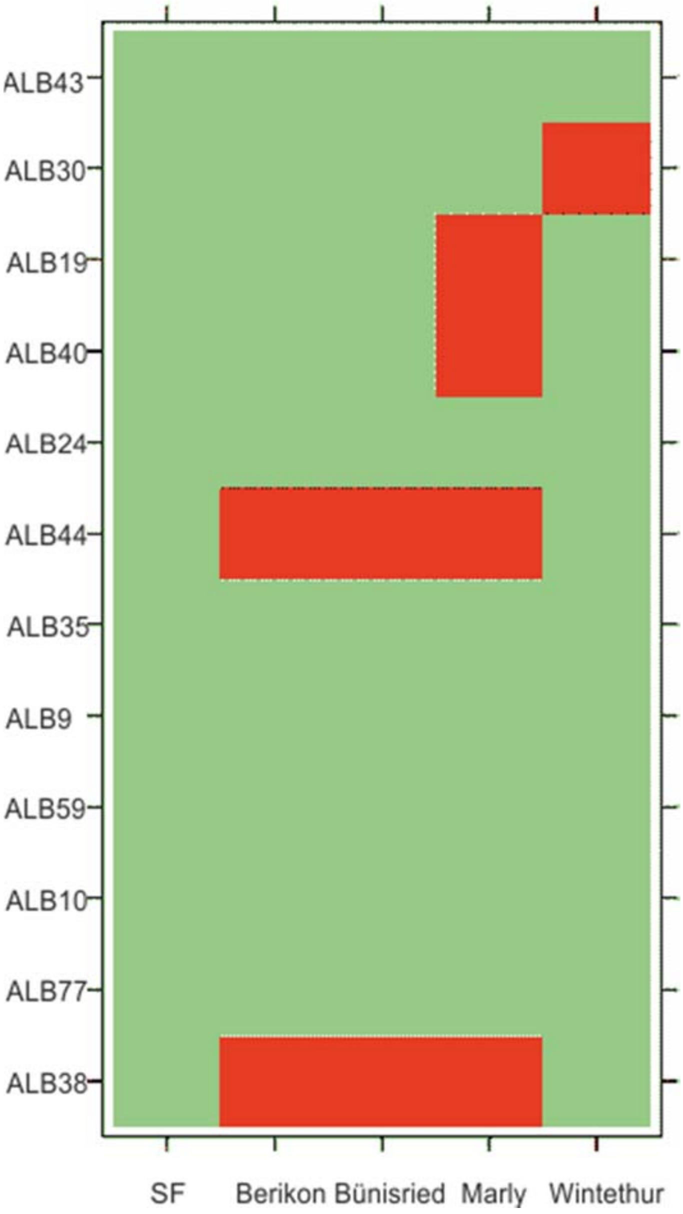

Figure S2. Pair-wise linkage disequilibrium among 13 SSR loci. Red color indicates *P* values < Bonferroni corrected threshold with α=0.05.

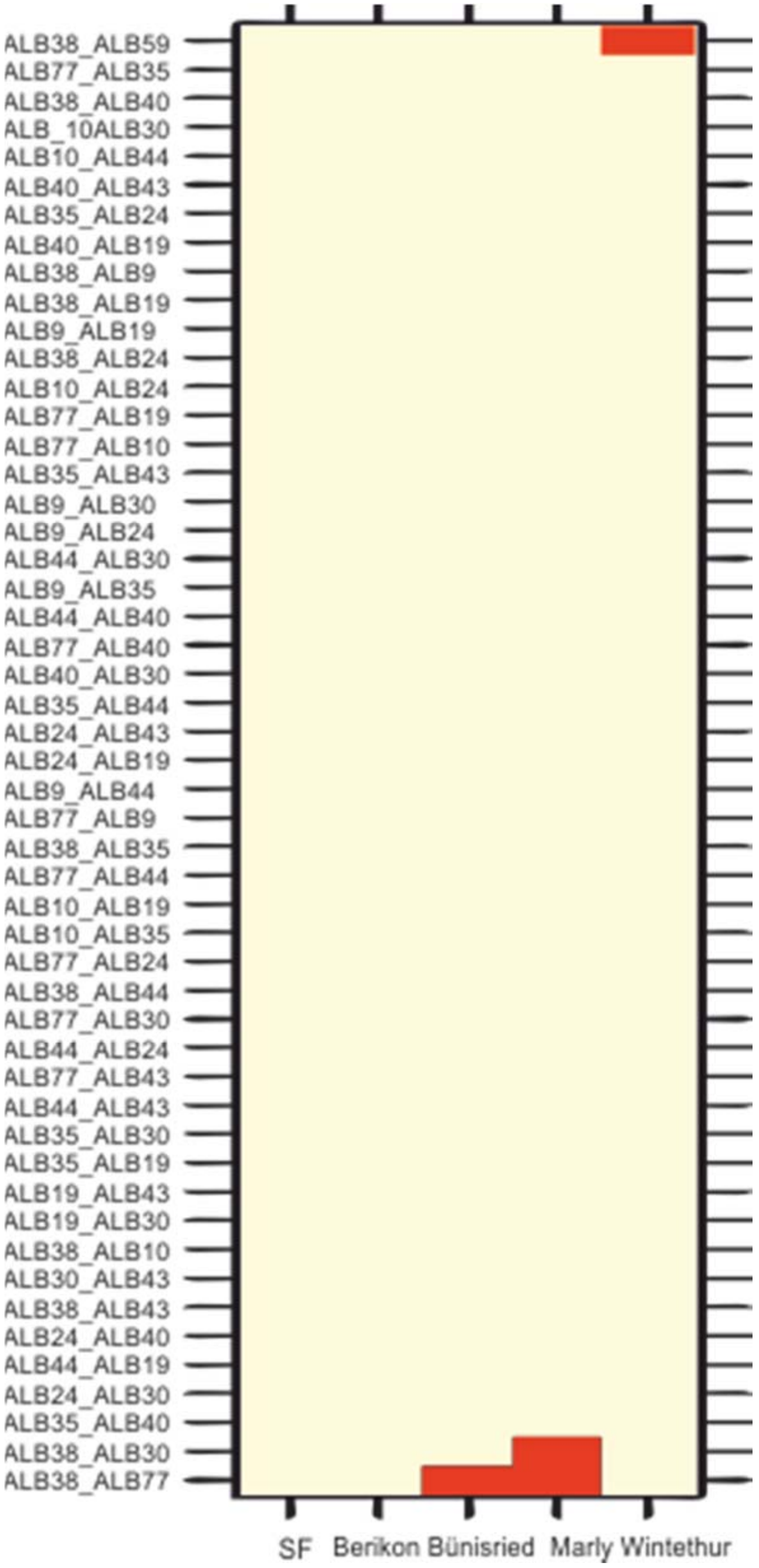

Figure S3. STRUCTURE bar plots using 13 SSRs of 192 ALB genotypes in four Swiss outbreaks and 7 single findings. Each bar represents the average estimated individual membership probability (ordinate) of an individual to belong to a specific cluster (indicated by specific color). In the lower left part (below), the barplots are supported by a scatterplot with mean log-likelihood values (± standard deviation) for different numbers of clusters (K) and in the lower right side with curve of ΔK .

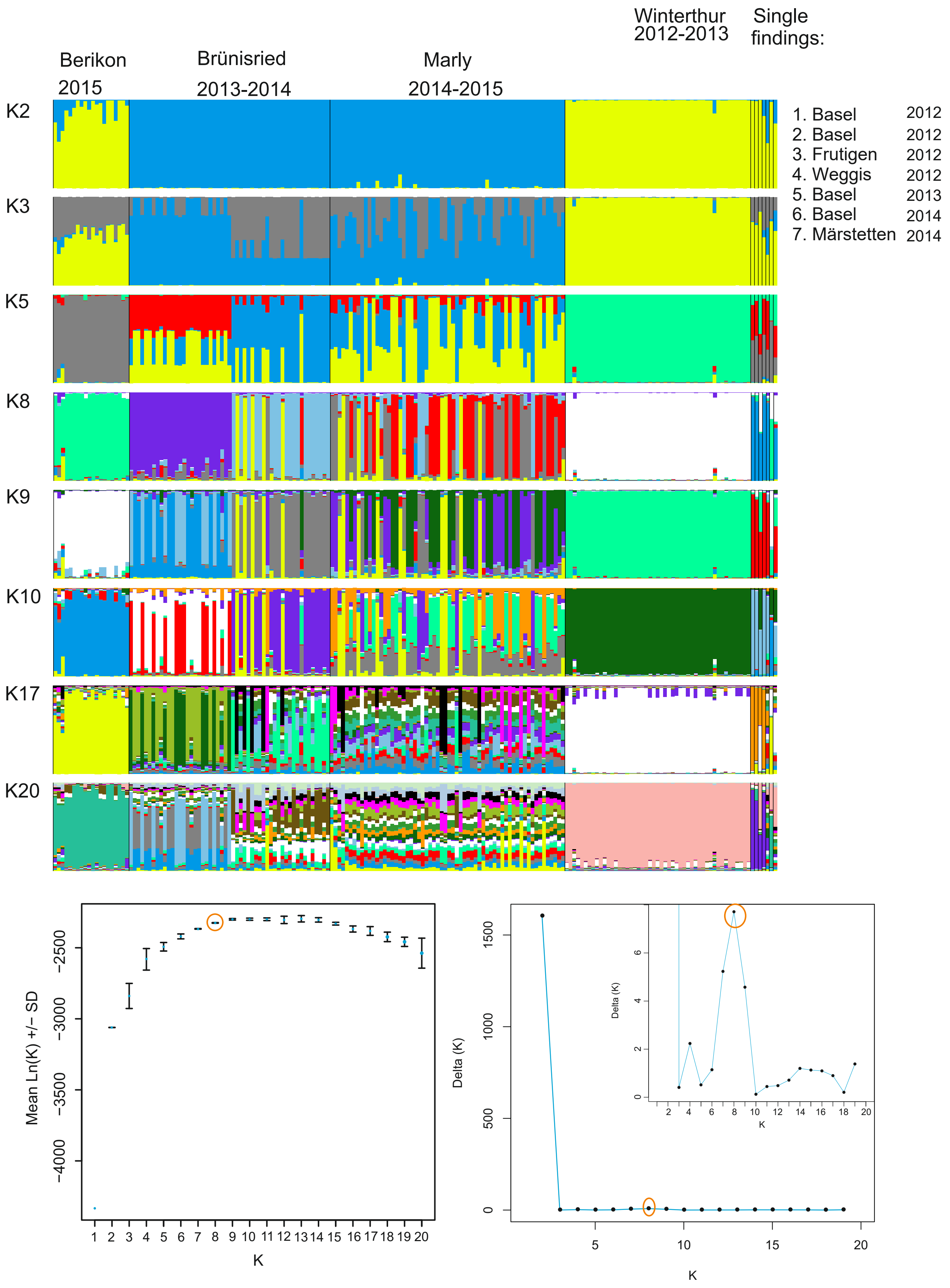

Figure S4. STRUCTURE bar plots using 13 SSRs of 101 ALB multilocus genotypes Marly and Brünisried outbreaks, canton Fribourg, Switzerland. Each bar represents the average estimated individual membership probability (ordinate) of an individual to belong to a specific cluster (indicated by specific color). In the lower left part (below), the barplots are supported by a scatterplot with mean log-likelihood values (± standard deviation) for different numbers of clusters (K) and in the lower right side with curve of ΔK .

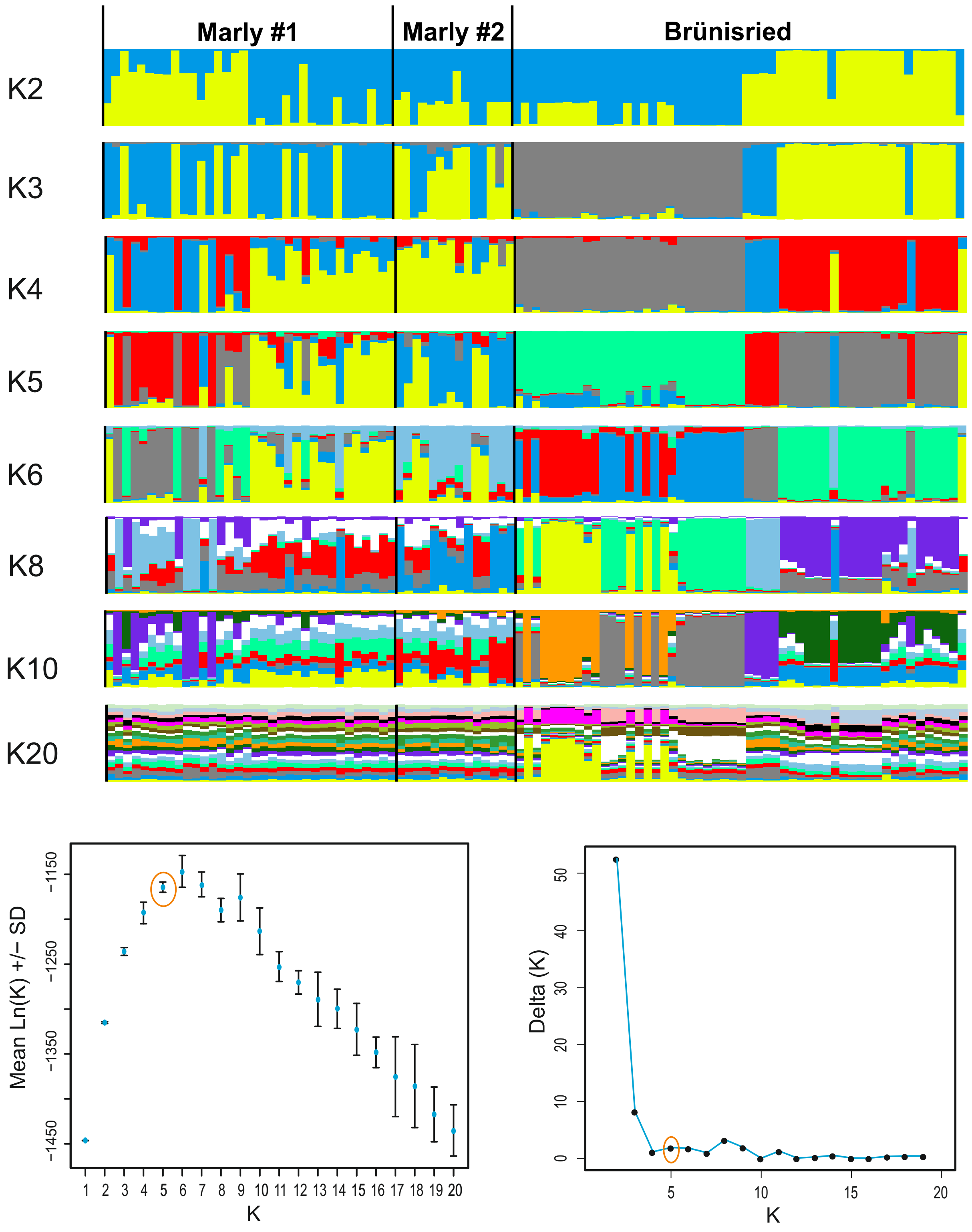

Details on the ABC analysis of Asian longhorned beetles outbreaks in Marly and Brünisried, Switzerland

Based on the ratio FST_Ma#1Br < FST_Ma#2Ma#1 < FST_Ma#2Br and on the STRUCTURE results, next 6 competing scenario of the demographic events were assumed (Figure S1).

In a first step, to define likelihood scenario of demographic events in Brünisried and Marly we simulated pseudo-observed datasets (PODs) under uniform distribution of the parameters in a broad ranges: 10 - 10000 of population effective size, from 1 to 10^4 generations of occurrence of the events according to the condition t3 > t2 > db2 > t1 > db1. In a second step, in order to infer the time in generation ago when particular demographic event happened in the selected in a first step the likelihood scenario we used more narrow time frame from 1 to 10 generation, consider information from the field observation and prior study of Forster & Wermelinger 2012. In this, second step we compared two scenario of the selected topology but excluding or including third change of effective size of initial population due to degradation (Figure S2). In addition, we estimated the posterior distributions of each demographic parameter for the best demographic model, by carrying out local linear regressions on the 1000 closest of simulated data sets, after a logit transformation to parameter values. In both steps, for each simulation, a value for each parameter was drawn from prior distribution and performed coalescent simulations with the same number of alleles and loci per population as in the observed dataset. A set of 15 summary statistics describing within and among population genetic diversity were calculated for each POD and the observed data. Population specific statistics included allele size variance (VAR), Garza -Williamson mean across loci (MGW). Between population statistics included F_{ST} , mean variance of the absolute allelic size (V2P), Goldstein's genetic distances between populations based on the variance in the repeats number (DM2). We used for simulation generalized stepwise-mutation model and default parameters of the DIYABC v.2.1.0.

The posterior probabilities of each competing scenario were estimated using a logistic regression on the 1% of 10^6 simulated datasets closest to the observed dataset. The best-fitting scenario was selected based on the highest posterior probability with a non-overlapping 95% confidence interval. We evaluated the ability of the ABC analysis to discriminate between the competing scenarios by analysing subset of 500 of closest to observed simulated data sets. Therefore, we estimated the Type-I error rate as the proportion of occurrences the best-supported scenario did not show the highest posterior probability among the competing scenarios and we estimated the Type-II error rate, by calculating the mean proportion of occurrences in which the best-supported model was incorrectly supported instead of selected for simulation one of the competing scenario.

Table S1. Mean posterior probabilities of the competing models for ABC analysis inferred from 1% of 10^6 simulated datasets.

First step							I type error	II type error
Post. prob. 0.95 CI	SC1	SC2	SC3	SC4	SC5	SC6		
*Direct	0.16 ± 0.01	0.14 ± 0.01	0.14 ± 0.01	0.16 ± 0.01	0.27 ± 0.03	0.13 ± 0.01	0.35	0.11
**LDA	0.05 ± 0.01	0.04 ± 0.01	0.03 ± 0.01	0.11 ± 0.01	0.65 ± 0.02	0.12 ± 0.03	0.32	0.15
Second step								
*Direct	0.61 ± 0.01	0.39 ± 0.01	-	-	-	-	0.06	0.29
**LDA	0.71 ± 0.04	0.29 ± 0.04	-	-	-	-	0.12	0.19

* Posterior probabilities inferred directly from summary statistics of the closest to observed simulated data sets; ** Posterior probabilities inferred from linear discriminants of the summary statistics of the simulated data sets

For the best supported scenario, the posterior probability distribution of time and demographic parameters were estimated, after a logit transformation, by local linear regression on the 1% of simulations closest to the observed data. Finally, for model checking we simulated 10000 PODs from the posterior under the best-supported scenario in order to evaluate whether this model could successfully reproduce the observed data.

Table S2. Parameter estimation for the likelihood scenario in natural units

Parameters	mean	madian	mode	Q2.5	Q5.0	Q25.0	Q75.0	Q95.0	Q97.5
*Ma1	5210	5310	8810	216	499	2700	7760	9520	9770
*Ma2	4620	4400	14	90	282	2050	7120	9410	9710
*Br	5300	5480	9570	86	364	2920	7890	9600	9820
**t1	7	7	8	4	5	6	8	9	9
**db1	6	6	6	4	4	5	7	9	9
*Ma1_b	185	102	36	20	25	52	234	668	791
*Ma2_b	65	25	13	11	11	16	51	265	473
*Br_b	123	60	31	13	17	33	122	503	746
**t2	10	10	10	8	8	9	10	10	10
*NA	2260	1490	850	213	300	797	3010	7170	8330

* - effective population size is provided in term of diploid individuals; ** - time of the event are provided in generation before present. Qx: x % quantile.

Table S3. Model checking of the likelihood scenario for ABC analysis

Summary stat.	Obs. value	Simulated
NAL_1_1	2.62	0.41
NAL_1_2	2.15	0.43
NAL_1_3	2.31	0.41
HET_1_1	0.34	0.43
HET_1_2	0.29	0.40
HET_1_3	0.27	0.34
VAR_1_1	0.87	0.52
VAR_1_2	2.79	0.87
VAR_1_3	4.45	0.93
MGW_1_1	0.61	0.03*
MGW_1_2	0.62	0.08
MGW_1_3	0.51	0.01*
N2P_1_1&2	2.62	0.35
N2P_1_1&3	2.77	0.39
N2P_1_2&3	2.54	0.39
H2P_1_1&2	0.35	0.43
H2P_1_1&3	0.31	0.36
H2P_1_2&3	0.29	0.35

Summary stat.	Obs. value	Simulated
V2P_1_1&2	1.51	0.68
V2P_1_1&3	4.00	0.90
V2P_1_2&3	4.82	0.94
FST_1_1&2	0.12	0.85
FST_1_1&3	0.08	0.55
FST_1_2&3	0.14	0.72
LIK_1_1&2	0.58	0.47
LIK_1_1&3	0.61	0.50
LIK_1_2&1	0.48	0.42
LIK_1_2&3	0.61	0.51
LIK_1_3&1	0.53	0.48
LIK_1_3&2	0.56	0.44
DAS_1_1&2	0.64	0.55
DAS_1_1&3	0.66	0.62
DAS_1_2&3	0.67	0.62
DM2_1_1&2	0.84	0.91
DM2_1_1&3	4.03	0.99**
DM2_1_2&3	4.45	0.99**

*, **, *** - corresponding to significant to tail-area probabilities (p-values) < 0.05, < 0.01 and < 0.001 respectively. Abbreviations for the summary statistics are as follows: mean number of alleles in population (NAL); mean expected heterozygosity in population (HET); mean size variance (VAR); mean Garcia-Williamson index (MGW); mean number of alleles pooling samples from populations (N2P); mean expected heterozygosity pooling samples from populations (H2P); mean variance of the absolute allelic size pooling samples from populations (V2P); F_{ST} between 2 samples (FST); mean individual assignment likelihoods of population i assigned to population j (LIK); shared allele distance between populations (DAS); dμ2 distance (DM2).

Figure S5. Six scenario of the demographic events and descriptions assumed for the first step of ABC evaluation. Time is not scaled on the schemes. Supported scenario marked with green rectangle.

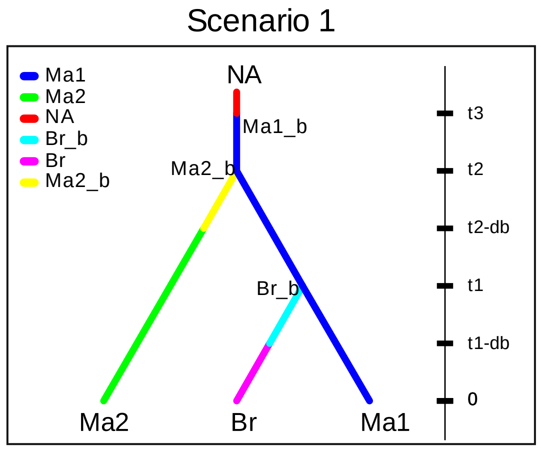

The initial ALB populations derived from ancestral NA population from South Korea and inevitably experienced bottleneck and reduction of the genetic diversity due to founder effect in time t3. Then in time t2 from population stepwise population Ma2_b was established, further developed to sampled Ma2. In the time t1 from Marly was transported fire wood with beetles to Brünisried and population Br_b was established, further on developed to sampled population Br.

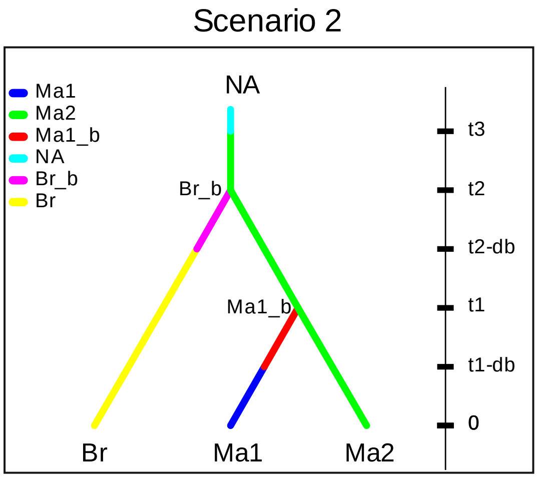

The initial ALB population Ma2 appeared from ancestral NA population, inevitably experienced bottleneck and reduction of the genetic diversity due to founder effect in time t3. Then in time t2 from population Ma2 was transported fire wood with beetles to Brünisried and population Br_b was established, further on developed to sampled population Br. In the time t1 from Ma2 stepwise population Ma1_b was established, further developed to sampled Ma1.

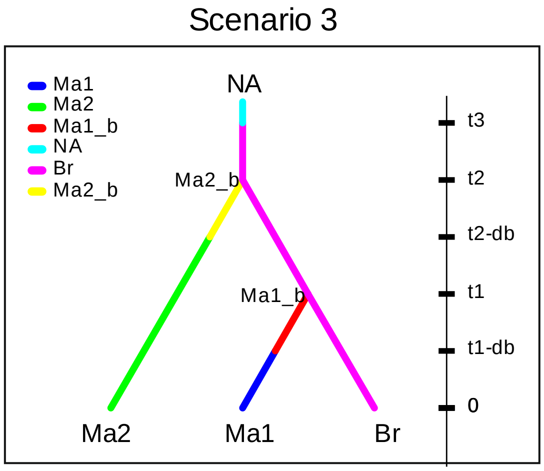

The infested wood from unsampled ancestral population NA were immediately transported to Brünisried and initial population Br was established in the time t3. Later on, in time t2 from population Br beetles were transported to Marly and population Ma2_b was established, which developed to sampled population Ma2. Then in t1 from Brünisried stepwise population Ma1_b was established, further developed to sampled Ma1.

Scenario 4

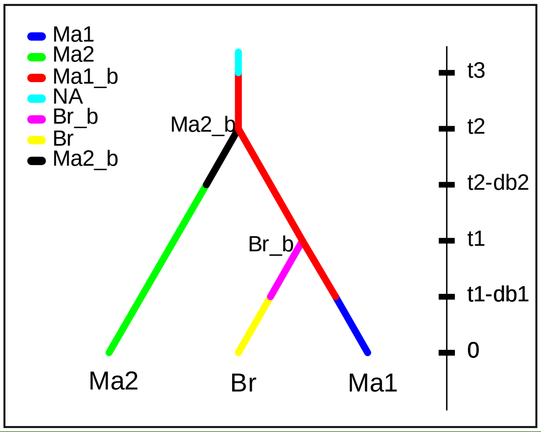

The initial ALB Ma1_b population derived from ancestral population NA to Marly in time t3. Inevitably experienced bottleneck and reduction of the genetic diversity due to founder effect. Then in time t2 from Ma1_b were infested one more location in Marly and Ma2_b founder population was established, later since time t2-db2 developed to sampled Ma2. In time t1 from Ma1_b was transported fire wood with beetles to Brünisried and initial bottlenecked population Br_b was established, further on developed to sampled population Br, whereas genetic drift and extinction of some lineages resulted in sampled population Ma1.

Scenario 5

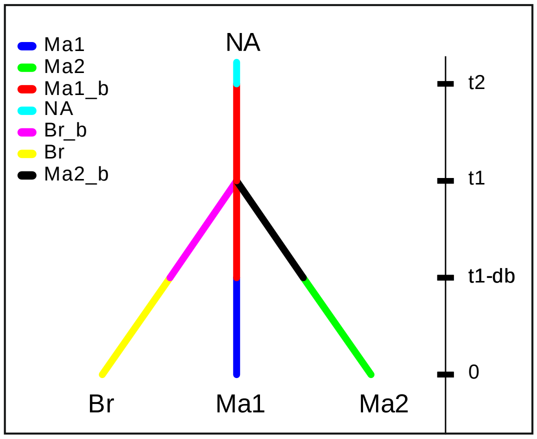

The initial NA population derived from ancestral population to Marly in time t2, experienced bottleneck and reduction of the genetic diversity due to founder effect and established as Ma1_b, with a time also experienced genetic drift and due to other natural demographic processes developed to sampled population Ma1. In time t1 from Ma1_b population was infested one more location in Marly, that is Ma2_b founder population, which developed to sampled population Ma2. Meanwhile, from Ma1_b was transported fire wood with beetles to Brünisried and initial founder population Br_b was established, further on developed to sampled population Br.

Scenario 6

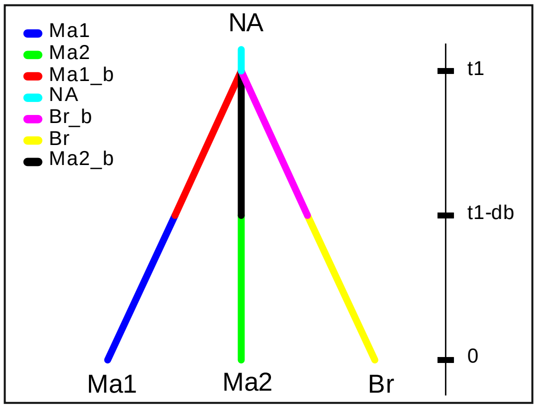

All three populations of ALB derived from ancestral population NA to both locations in Marly and to Brünisried approximately in the same time t1. Inevitably all experienced bottleneck due to founder effect and established initial populations Ma1_b, Ma2_b and Br_b. Then populations independently developed to sampled Ma1, Ma2 and Br.

Figure S6. Second step of ABC evaluation of the two assumed scenario. First scenario was supported in the first step of the analysis, whereas second scenario exclude population effective size change in Marly #1 in time t1-db. Supported scenario marked with green rectangle.

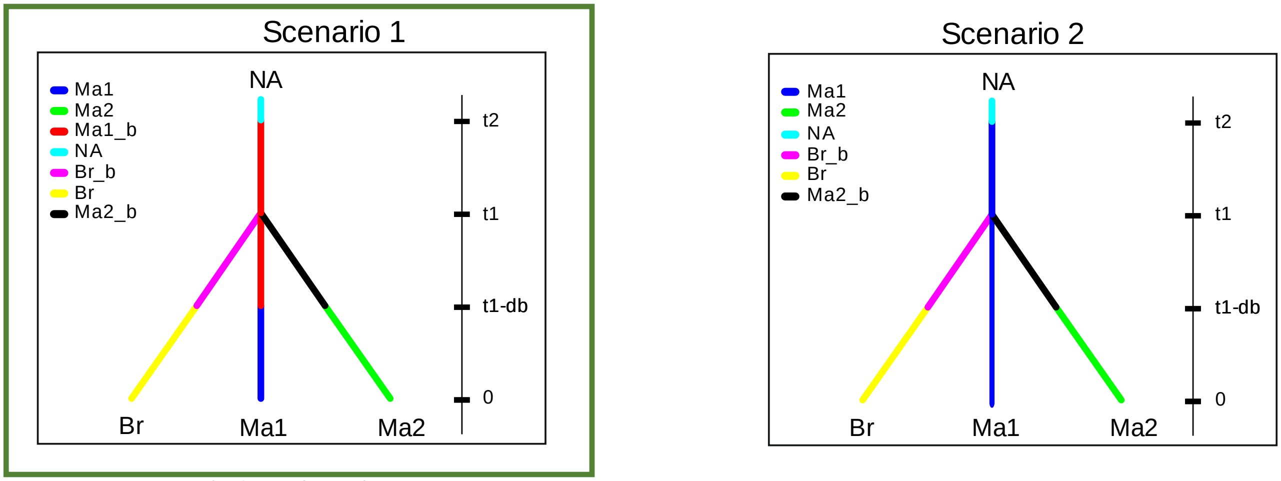

Time is not scaled on the schemes.
